# Supplementary material for: Malignant Pleural Mesothelioma: Preliminary Toxicity Results of Adjuvant Radiotherapy Hypofractionation in a Prospective Trial (MESO-RT)
Source: Cancers (Basel). 2023 Feb 7;15(4):1057. doi: 10.3390/cancers15041057 (PMC9954648; doi:10.3390/cancers15041057)
Supplement: Supplementary file 1 [file cancers-15-01057-s001.zip › cancers-2194226-supplementary.pdf]

Supplementary Materials - Raw data

Supplementary Table S1. Lung and other Organs at Risk (OAR) constraints.

	Total Lung					Contralateral Lung			
	NTCP (%)	V20 (%)	V5 (%)	Mean (Gy)		NTCP (%)	V20 (%)	V5 (%)	Mean (Gy)
**Mean**	13,11	32,72	40,56	17,71	**Mean**	0,456	0,454	3,502	2,109
**Max**	24,69	42,38	52,75	22,95	**Max**	0,477	1,41	6,03	2,47
**Min**	6,47	26,18	22,77	13,47	**Min**	0,41	0	1,46	1,29

	Other Organs at Risk						
	Spinal Cord Dmax (Gy)	Esophagus Mean Dose (Gy)	Liver Mean Dose (Gy)	Bowel Dmax (Gy)	Stomach Mean Dose (Gy)	Kidney OMO V18 (%)	Heart V30 (%)
**Mean**	17,8	15	9,94	31,07	7,01	6,4	14,48
**Std**	2,02	3,6	6,9	18,31	5,88	8,12	5,93
**Max**	22,85	22,07	24,29	56,13	30,52	37,8	25,78
**Min**	14,88	6,52	1,94	3,19	2,59	0	2,12

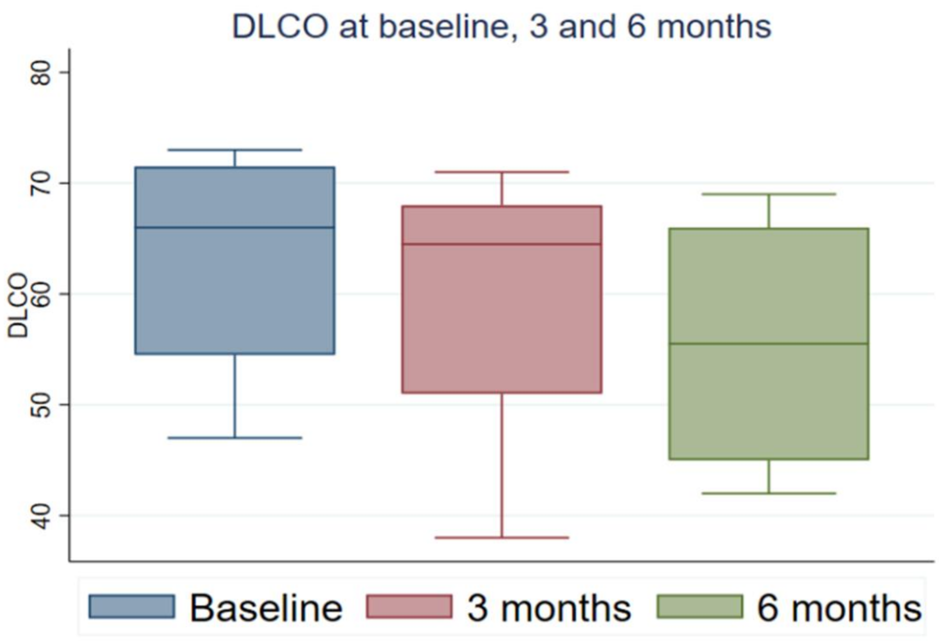

Supplementary Figure S1. Graph of DLCO at baseline, 3 months and 6 months after radiotherapy.

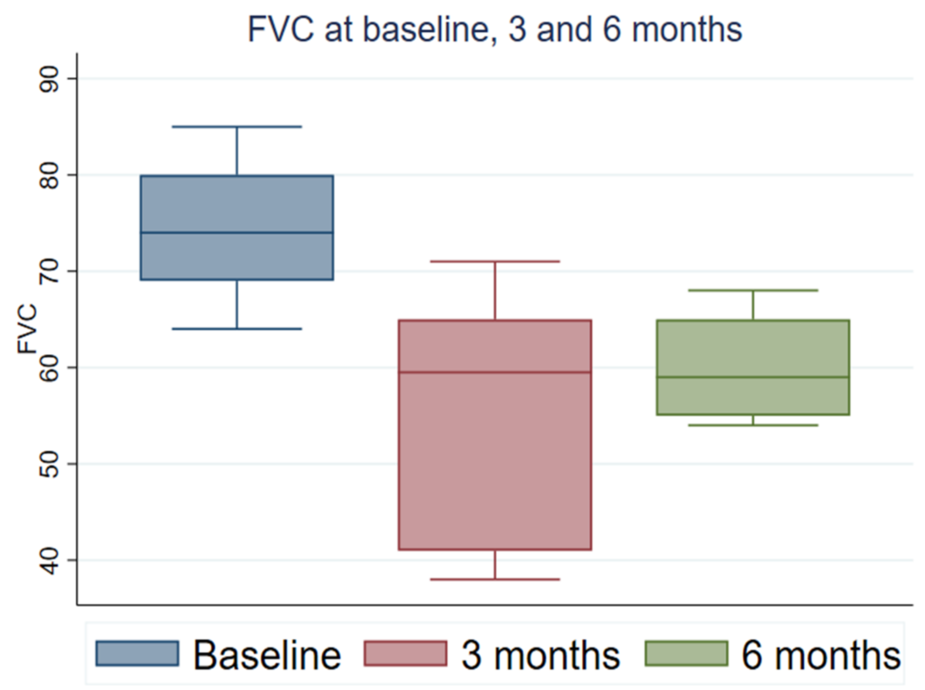

Supplementary Figure S2. Graph of FVC at baseline, 3 months and 6 months after radiotherapy.

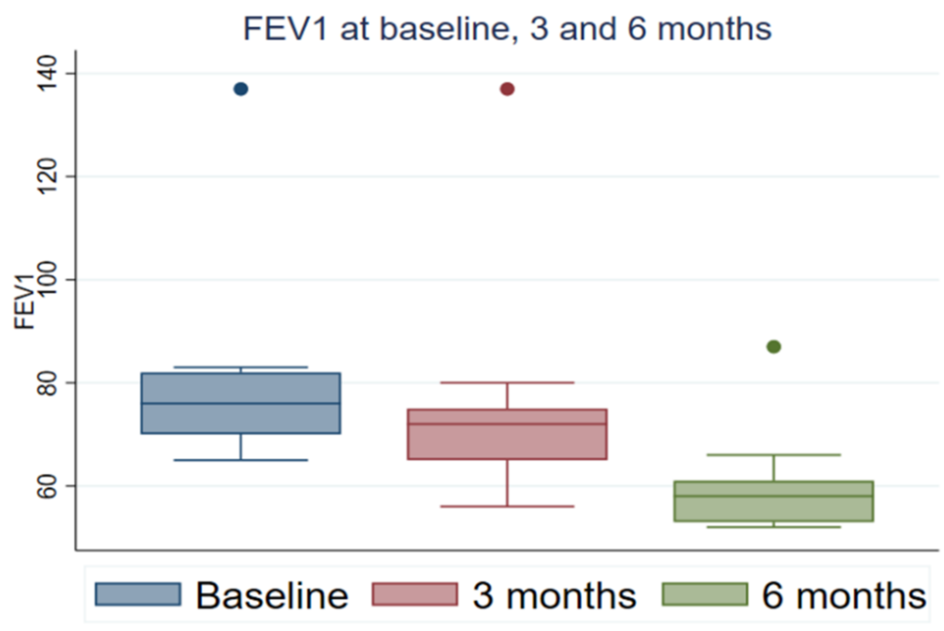

Supplementary Figure S3. Graph of FEV1 at baseline, 3 months and 6 months after radiotherapy.

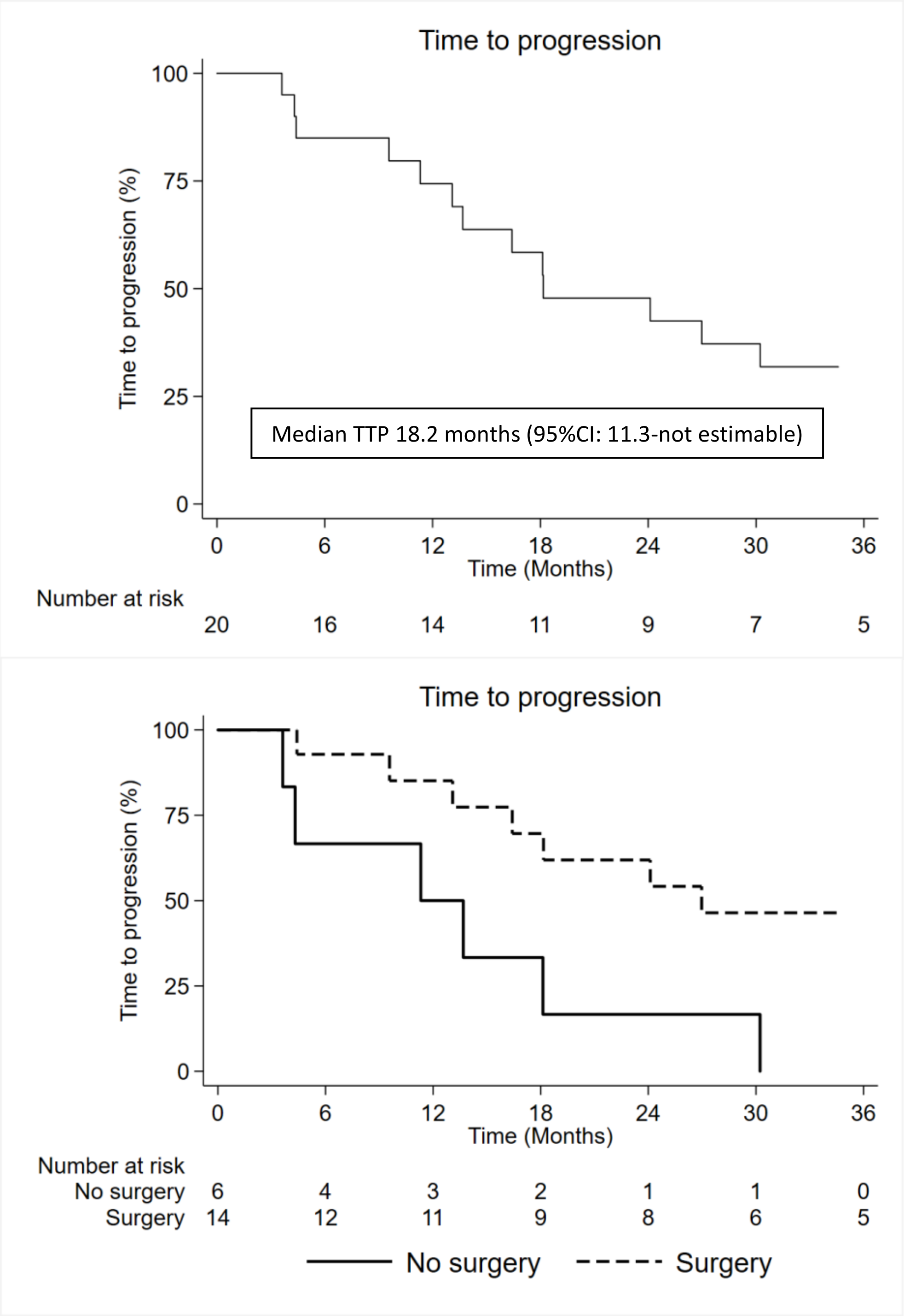

Supplementary Figure S4. (A) Graph of Time to Progression of all patients; **(B)** Graph of time To Progression of operated patients and no-operated patients.
